# Pelvic tenderness is not limited to the prostate in chronic prostatitis/chronic pelvic pain syndrome (CPPS) type IIIA and IIIB: comparison of men with and without CP/CPPS

**DOI:** 10.1186/1471-2490-7-17

**Published:** 2007-10-02

**Authors:** Richard E Berger, Marcia A Ciol, Ivan Rothman, Judith A Turner

**Affiliations:** 1 Departments of Urology, Rehabilitation Medicine and Psychiatry and Behavioral Sciences. The University of Washington, Seattle, Washington, USA

## Abstract

**Background:**

We wished to determine if there were differences in pelvic and non-pelvic tenderness between men with chronic prostatitis/chronic pelvic pain syndrome (CP/CPPS) Type III and men without pelvic pain.

**Methods:**

We performed the *Manual Tender Point Survey (MTPS*) as described by the American College of Rheumatology on 62 men with CP/CPPS Type IIIA and IIIB and 98 men without pelvic pain. We also assessed tenderness of 10 external pelvic tender points (EPTP) and of 7 internal pelvic tender points (IPTP). All study participants completed the National Institutes of Health Chronic Prostatitis Symptom Inventory (NIH CPSI).

**Results:**

We found that men with CPPS were significantly more tender in the MTPS, the EPTPS and the IPTPS. CPSI scores correlated with EPTP scale but not with IPTP scale or prostate tenderness. Prostatic tenderness was present in 75% of men with CPPS and in 50% of men without CPPS. Expressed prostatic fluid leukocytosis was not associated with prostatic tenderness.

**Conclusion:**

Men with CP/CPPS have more tenderness compared to men without CPPS. Tenderness in men with CPPS is distributed throughout the pelvis and not specific to the prostate.

## Background

Idiopathic prostatitis (also called non-bacterial prostatitis) and prostatodynia have been renamed by a National Institutes of Health (NIH) consensus panel to Chronic Prostatitis/Chronic Pelvic Pain Syndrome (CP/CPPS) Types IIIA and IIIB, respectively. These syndromes are characterized by pelvic pain, a negative prostatic localization culture, and no specific diagnosis accounting for the pain. Voiding symptoms may or may not be present. Type IIIA is differentiated by leukocytes in the expressed prostatic secretions (EPS) and Type IIIB is characterized by a lack of inflammation in the EPS [1]. Recently, a large multicenter study found that the symptoms of prostatitis were not associated with prostatic inflammation, casting doubt on prostatic inflammation as the direct cause of the pain[2].

Pelvic tenderness has not been investigated in normal men. In CP/CPPS, tenderness of the prostate is often present [3,4], however its relationship to prostatic inflammation has not been investigated. Whether pain is localized to the prostate or is part of a more generalized tenderness has not been determined.

The quantification of tenderness is difficult and subject to considerable inter-rater variability[5,6]. However, tenderness is extremely important in everyday clinical evaluation and is routinely used to identify pathological processes. The American College of Rheumatology has developed *The Manual Tender Point Survey (MTPS) *as a standardized method to evaluate fibromyalgia (FM)[7]. This instrument includes 18 examination points that are often tender and 3 control points that are infrequently tender in patients with FM. Fibromyalgia (FM)is distinguished by multiple tender sites and pain in four quadrants of the body[8]. Patients with interstitial cystitis (IC), a condition that may be related to CP/CPPS, have been found to have increased tenderness in the pelvis and and in the MTPS as compared with patients without IC[9]. The purpose of this study was to use the MTPS, expanded to include pelvic tender points, to test the hypothesis that men with CP/CPPS would show more overall tenderness than men without pelvic pain. We hypothesized that the location of tenderness in CP/CPPS may indicate the location of underlying pathology and the extent of the tenderness may indicate a localized or more systemic nature of the syndrome. We explored the relationship of prostatic secretion inflammation to tenderness. A finding of an association between prostatic inflammation and prostatic tenderness would support the possibility that inflammation produces tenderness and pain. More muscle tenderness in men with CP/CPPS IIIB than in men with CP/CPPS IIIA, as suggested by Segura[10], would support the continued distinction between the two syndromes. We also hypothesized that there would be a relationship between tenderness and CPSI scores.

## Methods

### Study participants

The University of Washington's institutional review board approved the study reported here as part of a more comprehensive study of men with pelvic pain. All subjects signed written consents explaining study procedures. Other findings from the larger study have been reported previously[11-16]. Patients with CP/CPPS were identified from the University of Washington Prostatitis Clinic. Study inclusion criteria for CP/CPPS patients were age 18–65 years, diagnosis of CP/CPPS Type IIIA or IIIB made by a urologist at the clinic, negative prostatic localization cultures for pathogens, pelvic pain of at least 3 months duration, and no other identified pathology to account for symptoms. Controls were healthy volunteers without pelvic pain or history of any urologic disease, recruited from advertisements. Controls were not excluded for the presence of pain in other areas of the body. Controls were paid $250.00 for participation in the study, but patients were not paid. Evaluators were not blinded as to the patient or control status of the subjects.

### Procedures

Patients and controls were evaluated during a screening visit to the clinic. Study participants were requested not to take anti-microbial agents within six weeks of examination and were instructed to abstain from ejaculation for 48 hours prior to their appointment. All subjects provided demographic information on questionnaires and the NIH Chronic Prostatitis Symptom Index (CPSI), a measure of pain severity, urinary symptoms and quality of life[17,18]. Following a standardized history and physical examination, patients and controls underwent four-glass urine localization cultures and urethral cultures for *C. trachomatis, U. urealyticum, M. hominis*, and *T. vaginalis*. Expressed prostatic secretions (EPS) were assessed for leukocyte concentration by hemocytometer counts using a phase contrast microscope[14]. We defined CP/CPPS Type IIIB as less than and Type IIIA as more than 500 leukocytes/microliter[11]. Patients and controls were excluded from further study participation if any of the following conditions were present: active urinary tract infection or infection localized to the prostate from a four-glass urine sample, positive cultures for *C. trachomatis or N. gonorrhoeae*, genitourinary malignancy, evidence of suicidal ideation or psychosis, post-surgical pain, pain from another source in the genitourinary tract (e.g., renal calculi), history of radiation therapy, or history of genitourinary tuberculosis.

### Tenderpoint assessment

**Figure 1:**
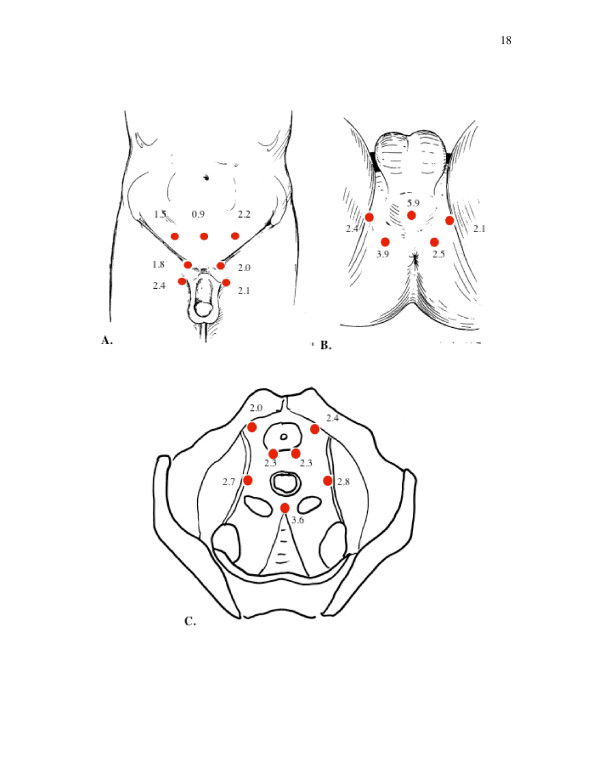
Diagram of pelvic tender points used in this study. B) Ventral depiction of EPTPS points, C) Perineal view of EPTPS, 4) Horizontal view of IPTPS. Numbers represent ratio of patient to control number of positive tender points and therefore relative tenderness of patients over controls The red dots indicate the location of tender points. The points over the adductor insertions are represented on ventral and perineal depictions.

The MTPS was performed by applying with a thumb 4 kg of pressure to each of the 18 tender and 3 control points (Fig 1A). The subject is asked to rate the tenderness experienced on a scale of 0 (no pain) to 10 (worst pain ever experienced). The diagnosis of FM requires tenderness for ≥ 11 of the 18 points[7]. Because the MTPS does not include the pelvis, we added an external pelvic tender point scale (EPTPS) consisting of 10 tender points and an internal pelvic tender point scale (IPTPS) consisting of seven tender points including the prostate. (Fig. 1A, B and 1C). These pelvic points were determined based on the clinical experience of one of the authors (R.B.). The EPTPS included bilateral points over the pubic symphysis, mid-inguinal canal, crura of the penis in the perineum, and the adductor tendons at the attachment to the pelvis. Midline points tested were suprapubically 4 cm above the symphysis and over the bulbar urethra in the mid-perineum. The IPTPS included bilateral points over the prostate midway between the base and apex, the pubic symphysis and endopelvic fasica on either side of and just lateral to the prostate, and the lateral pelvic sidewalls over the levator muscles. A single point midway between the rectum and the sacrum was also tested posteriorly (Fig 1C). For the MTPS tender point and control sites, EPTPS, and IPTPS, the number of tender points was calculated and a severity score was calculated by adding the individual ratings. Prostatic scores (0–10) were taken from the left and right prostate and added together (maximum of 20) for the total prostatic score.

Each of the three examiners (two urologists and one nurse practitioner; R.E.B, J.C.L., I.R.) practiced until he could apply 4 ± .25 kg ten times in a row on a "Chatillon" dolorimeter.(Amtek, Largo, FL.) Study participants were examined with the MTPS[7], then the EPTPS, then the IPTPS and prostate. Each examiner recalibrated the force of his finger at least weekly. Examiners 1, 2, and 3 examined 18, 32, and 48 control subjects, and 7, 33, and 22 pain patients, based on logistical scheduling considerations.

A few pain patients and controls had missing tenderness measure values for some points (but not all) because of omissions in record keeping. The three examiners examined patients comparable in symptom severity according to the NIH CPSI scores (p = 0.996, Kruskal-Wallis test).

### Statistical analyses

Descriptive analysis was performed for all variables in the study. Differences between pain patients and controls in demographic characteristics were assessed by t-tests for age and Chi-square tests for all other variables. Despite the training protocol, there were some systematic differences between examiners in tender point examination scores. Given that the distributions of the tender point measures were not normal, we used two complementary approaches to examine differences between groups while accounting for examiners. First, we dichotomized each tenderness scale severity score (lower 10% of the possible scores, highest 90% of possible scores; Table 1). We then applied the method of Mantel-Haenszel to compare the odds that a pain patient score is in the highest 90% of the scale, to the odds of a control subject, while controlling for the examiner. ^19^Second, we analyzed the original scale of severity scores separately for each examiner using the Mann-Whitney test to compare controls to pain patients. This analysis was based on the premise that if there were differences between controls and pain patients, the difference would be seen within the group of subjects examined by each examiner. Consistency in significant differences would lead to the conclusion that the two groups of subjects are different regarding the tenderness scales, although we cannot specifically state the magnitude of that difference across clinicians.

**Table 1:** Definition of the dichotomous transformation of the tender point examination scale severity scores

		Dichotomous variable defined as	
		
Scale	Possible Range	0 if value in range	1 if value in range
MTPS Control	0–30	0–3	4–30
MTPS Tender points	0–180	0–18	19–180
EPTPS	0–100	0–10	11–100
IPTPS	0–50	0–5	6–50
Total Prostate Score	0–20	0–2	3–20

MTPS = myofascial tender point score, EPTPS = external pelvic tender point score, IPTPS = internal pelvic tender point score, Total Prostate Score = sum of scores of left and right prostate.

We calculated Spearman correlations to examine the association between the tenderpoint scores and the NIH CPSI. Inflammation (defined by EPS leukocytes count) was compared to tenderness (as defined in Table 1) by Chi-square test.

## Results

Seventy-two men with CP/CPPS and 98 controls were enrolled as part of a larger study[11-16]. The tender points examination was instituted after the first 10 CP/CPPS subjects. Therefore, the present report is based on 62 CP/CPPS subjects and 98 controls. Patients and controls differed significantly on age (p < 0.001), education (p < 0.001), and employment status (p = 0.02), but not on race (p = 0.50) and marital status (p = 0.09) (Table 2). Among the CP/CPPS patients, 40.3% were Type IIIA, 18.1% Type IIIB, and 41.7% had undetermined type because EPS could not be obtained. Among controls, 38.8% had leukocyte counts > 500/mm^3^and 26.5% = 500/mm^3^, and EPS was not obtained for 34.7%. Subjects who the examiner was able to obtain EPS did not differ demographically from those in whom the examiner was unable to obtain EPS.

**Table 2:** Sample Demographic Characteristics and NIH CPSI Scores

	Pain Patients n = 62	Controls n = 98	P-value*
Age, years, mean (SD)	40.7 (10.4)	34.2 (10.4)	< 0.001
Race			
Caucasian (%)**	88.5	84.7	0.50
Marital Status, % **			
Married/living with significant other	62.9	44.9	0.09
Divorced/separated	8.1	12.2	
Never married	29.0	42.9	
Education, %**			
Some HS, HS/GED, or Vocational/Technical	18.3	3.1	< 0.001
Some college	6.7	28.6	
College graduate	36.7	36.7	
Graduate/professional school	38.3	31.6	
Employment, %**			
Full time work	72.1	55.7	0.02
Part-time work	8.2	18.6	
School (full or part-time)	8.2	20.6	
Retired, homemaker, unemployed	11.5	5.2	
NIH Chronic Prostatitis Symptom Index***			
Total Score, mean (SD)	21.8 (6.9)	0	
Urinary Symptoms, mean (SD)	4.0 (2.8)	0	
Pain, mean (SD)	10.1 (4.0)	0	
Quality of Life, mean (SD)	7.6 (2.6)	0	

* Difference in mean age was tested using t-test. All other differences were assessed by a Chi-square test.

**Information was not available on race for 1 pain patient, on education for 2 pain patients, and on employment for 1 pain patient and 1 control.

*** NIH CPSI data for pain patients were missing for 9 patients on the total score, 5 on the Urinary Symptoms scale, 8 on the Pain scale, and 5 on the Quality of Life scale. Control subjects were eligible for the study only if their NIH CPSI score was zero.

The proportion of subjects in the higher category of pain level for each tenderness site is shown in Table 3. Except for the control sites for tenderness, the pain group had consistently larger proportions of subjects with high scores than the control group. For example, 28.7% of the controls and 67.3% of the pain patients had high scores (as defined in Table 1) for IPTPS. Tenderness was found on prostate examination in 28 of 94 (29.8%) controls and 31 of 54 (57.4%) CP/CPPS patients.

**Table 3:** Proportion of high scores (according to definitions in Table 1) for control and pain subjects

	Proportion of High Score (# missing)	
	
Scale	Controls (n = 98)	Pain Patients (n = 62)
MTPS Control	5.1 (0)	3.2 (0)
MTPS Tender points	11.8 (5)	16.7 (2)
EPTPS	18.4 (11)	49.1 (7)
IPTPS	28.7 (4)	67.3 (7)
Prostate Score	29.8 (4)	57.4 (8)

To study the difference in proportions adjusting for clinicians, the dichotomized variables (low versus high level of pain) were analyzed using the Mantel-Haenzel method (Table 4). Pain patients were more likely than control subjects to be in the higher category of pain level for EPTPS, IPTPS, and prostate severity scores (odds ratios = 9.59, 6.25, and 4.98; p < 0.0001 for all), but not for the control site score (p = 0.98). There was a trend towards higher odds of pain patients being in the higher pain category for the MTPS tender points score (p = 0.069). When we examined dichotomized pain scores every point tested in the pelvis was more often painful in CPPS patients than controls except the suprapubic point. Relative ratios of painful points in CPPS patients to controls were highest in the mid perineum at 5.9. Most other ratios were 2–3. (Fig 1)

**Table 4:** Common Odds Ratio Estimates (Mantel-Haenszel Method) for Examiners in Dichotomized Tenderness Severity Scores, Controlling for Differences across examiners.

Scale	Estimated Odds Ratio*	Asymptotic 95% Confidence Interval	P-value for Odds Ratio
MTPS Control	0.98	0.17 – 5.67	0.98
MTPS Tender points	2.64	0.93 – 7.51	0.069
EPTPS	9.59	3.40 – 27.06	< 0.001
IPTPS	6.25	2.87 – 13.61	< 0.001
Prostate Score	4.98	2.21 – 11.24	< 0.001

* Ratio of the odds that a case has a score in the highest 90% by the odds that a control has a score in the highest 90%.

The second approach used the originally observed scores without dichotomization. In comparisons of the median scale scores of the patient and control groups for each examiner, there were statistically significant differences for all scales except the control sites (Table 5). For examiner 1, there were no statistically significant differences, although there were trends towards significant differences on the IPTPS and prostate scales, most likely because this clinician saw the smallest number of pain patients and controls.

**Table 5:** Median scores by group and examiner.

Variable	Examiner	Median (Range)		P-value for Mann-Whitney test
			
		Controls	Pain Patients	
MTPS Control (0–30)	1	1.0 (0–8)	2.0 (0–5)	0.76
	2	0.0 (0–0)	0.0 (0–2)	0.16
	3	0.0 (0–6)	0.0 (0–4)	0.54
MTPS Tender points (0–180)	1	6.0 (0–56)	23.0 (0–52)	0.26
	2	0.0 (0–3)	1.0 (0–17)	**0.007**
	3	1.0 (0–39)	9.0 (0–36)	**0.013**
EPTPS (0–100)	1	15.0 (0–39)	26.0 (0–43)	0.43
	2	0.0 (0–8)	1.0 (0–56)	**0.04**
	3	1.0 (0–37)	14.5 (0–49)	**< 0.001**
IPTPS (0–50)	1	5.5 (0–26)	19.0 (0–40)	**0.051**
	2	0.0 (0–31)	8.0 (0–49)	**< 0.001**
	3	2.0 (0–19)	14.0 (0–34)	**< 0.001**
Prostate Score(0–20)	1	2.5 (0–11)	6.0 (0–19)	0.09
	2	0.0 (0–11)	.5 (0–20)	**0.001**
	3	1.0 (0–10)	7.0 (0–20)	**< 0.001**

Additionally, Spearman correlation was calculated for the tenderness points scores and the NIH CPSI for the pain patients. All tender point scales were statistically significant at 0.001 level, varying from estimated correlations of r = 0.29 (IPTPS and Control) to 0.73 (IPTPS with prostate score). The NIH CPSI Pain scale was correlated significantly with the FM and EPTPS, but not the IPTPS or prostate score suggesting that the CPSI pain score is more related to external than internal tenderness. However, plots of NIH CPSI versus each scale (graphs not shown here), showed that the associations are not necessarily linear, and therefore, they have limited value in describing the association between the variables.

EPS was obtained for 64 controls and 40 patients. Inflammation was defined as having leukocyte counts > 500/mm^3 ^in the EPS sample. There was no association between inflammation in EPS and the dichotomous prostatic tenderness (p = 0.42) or FM, IPTPS, and EPTPS dichotomous variables (all p-values > 0.23).

## Discussion

Prostatic tenderness has been described in men with prostatitis and CP/CPPS and is considered to be a characteristic of both CP/CPPS Type IIIA and IIIB[4]. However, the findings of this study indicate that not all men with CP/CPPS have prostate tenderness, and about 30% of men without CP/CPPS have such tenderness. Tenderness in the internal and external pelvis as well as extra-pelvic regions in men with CP/CPPS has not been described. To our knowledge, this is the first study that has demonstrated that men with CP/CPPS have increased tenderness in FM tender points and in specific internal and external non-prostatic pelvic locations. For example, men with CP/CPPS were 9.59 and 4.98 times more likely than men without pelvic pain to have scores in the higher 90% of the EPTPS and prostate scales, respectively. This suggests that CP/CPPS Type III is a pan-pelvic pain syndrome and that tenderness is not limited to the prostate. For each point in the pelvis that we tested, the CPPS group had more tenderness than the control group. Furthermore, increased tenderness even extended outside of the pelvis to FM points suggesting a systemic component. The labeling of chronic pelvic pain in men as "prostatitis" may well mislead both patients and physicians into thinking that the syndrome has a more limited focus and etiology than it actually may have[20].

The finding of increased tenderness in MTPS tender points in men with CP/CPPS is in accord with findings of diffusely increased tenderness in women with interstitial cystitis, a syndrome possibly related to CP/CPPS[9]. We hypothesize that central and/or peripheral pain sensitization in the pelvis may account for the diffuse symptoms and tenderness found in pelvic pain syndromes[21-25]. We have previously shown that there is sensitization to perineal heat sensation in some men with CP/CPPS[12,26] and that men with CP/CPPS often have abnormalities of pelvic and abdominal muscular function and sensation [13]. The diffuse tenderness on pelvic examination in our present study may be a manifestation of mechanical sensitization to pressure with the development of allodynia and hyperesthesia mediated via the CNS. If prostatic inflammation was the source of prostatic pain and the non-prostate pelvic tenderness was secondary to muscle guarding, we would have expected to find a relationship of prostatic secretion inflammation to prostatic and muscle tenderness. Since we found no such associations, we hypothesize that prostatic and other pelvic tenderness may both be related to an another more dominant process such as central or peripheral neural sensitization and that inflammation in prostatic secretion may be incidental.

Limitations of this study should be acknowledged. The sample came from a university tertiary care population and the study findings may not generalize to other populations. The control group was a convenience sample of volunteers and could have selection bias. Controls were younger, and although we found no relationship of tenderness scores to age, other unknown differences may have contributed to the differences found between patients and controls in examination findings. Furthermore, we did not assess test-retest stability of the tender point examination scores, and there were interrater differences. The determination of tenderness is a standard part of clinical examination, although variation from examiner to examiner is well known clinically and experimentally[5,6]. Although some of our examiners consistently found more tenderness, each examiner separately found more tenderness in CPPS patients in the areas examined.

## Conclusion

We found that men with CP/CPPS have generalized internal and external pelvic tenderness. The pathophysiology of CP/CPPS involves the entire pelvis and not only the prostate. Our findings suggest that further research involving the assessment of intra-and extra-pelvic tender points may prove fruitful in increasing scientific understanding of, and developing more effective treatments for, male chronic pelvic pain syndromes.

## Abbreviations

MTPS-Manual tender point scale

IPTPS-Internal pelvic tender point scale

EPTPS-External pelvic tender point scale

CP/CPPS-Chronic prostatitis/chronic pelvic pain syndrome

NIH CPSI-National Institutes of Health Chronic Prostatitis Symptom Index

## Competing interests

The author(s) declare that they have no competing interests.

## Authors' contributions

REB: Principal investigator for study. Designed and carried out study.

MEC: Assisted with design, performed statistical analysis, reviewed manuscript.

IR: Performed study procedures and reviewed manuscript.

JAT: Assisted with design, analysis and manuscript review.

All authors have read and approved the final manuscript.

## Pre-publication history

The pre-publication history for this paper can be accessed here:


